# Healthcare services for people with acquired disability in South-East Queensland, Australia: Assessing potential proximity and its association with service obstacles

**DOI:** 10.1016/j.ssmph.2022.101209

**Published:** 2022-08-17

**Authors:** David N. Borg, Joshua J. Bon, Michele M. Foster, Ali Lakhani, Melissa Kendall, Timothy Geraghty

**Affiliations:** a The Hopkins Centre: Research for Rehabilitation and Resilience, Menzies Health Institute Queensland, Griffith University, Nathan, Brisbane, Australia; b School of Health Sciences and Social Work, Griffith University, Brisbane, Australia; c Australian Research Council Centre of Excellence for Mathematical and Statistical Frontiers, Brisbane, Australia; d School of Mathematical Sciences, Queensland University of Technology, St Lucia, Brisbane, Australia; e La Trobe University, Melbourne, Australia; f Division of Rehabilitation, Princess Alexandra Hospital, Metro South Health Hospital and Health Service, Brisbane, Australia

**Keywords:** Access, Brain injury, Spinal cord injury, Rehabilitation

## Abstract

This study described access potential in South-East Queensland, to healthcare services commonly used by people with acquired disability; and investigated the association between service proximity and perceived service obstacles. First, we described accessibility by conducting a spatial analysis to create maps of potential accessibility to health services in South-East Queensland. Queensland statistical area level 2 (SA2) locations were combined with the residential locations of participants from a longitudinal cohort study involving people with ABI and SCI. The locations of selected health services of interest were identified from Health Direct's National Health Service Directory. Travel times via motor vehicle were modelled with Robust Gaussian Stochastic Process, to smoothly interpolate between the sparse time-to-service observations. Probabilistic predicted values were generated from the model and were used to construct service accessibility maps of South-East Queensland. Disability population data were used to identify SA2s with relatively low service access but a high disability population. Second, we examined perceived service obstacles, by investigating the relationship between potential access to services and perceived service obstacles was examined using data from 63 people with ABI or SCI discharged from the specialist state-wide rehabilitation services, located in South-East Queensland. Obstacles to accessing service in relation to resource availability, transportation and finances were collected three-months after discharge, using the Service Obstacle Scale. Travel times to the closest health service were computed for each individual and were compared to their Service Obstacle Scale responses. Access potential was highly variable, particularly for allied health services. We identified several low-access, high-disability population areas. These hotpots of poor access were generally to the north and west of greater Brisbane. Longer travel times to allied health services were associated with 260% higher odds of agreeing that resource availability was an obstacle to accessing services. Policy makers should be concerned with the hotspots of poor access identified.

## Highlights

• There was considerable variability in access potential, which was generally greater for allied health than medical specialist services.
• We highlighted low-access high disability population areas, which were generally in outer suburbia.
• Poorer potential access to allied health services was associated with resource availability being rated as an service obstacle.
• We did not find any evidence that proximity to allied health services was associated with transportation or finance obstacles.

## 1 Introduction

Despite increased attention from policy makers and advocates over the past decade, access to health and rehabilitation services for people with disability remains a global concern (World Health Organization, 2011). The gravity of the situation was brought to international attention by The World Health Organization's (WHO) first *World Report on Disability* (World Health Organization, 2011), which highlighted the poor access and high unmet needs experienced by people with disability, compared to the general population. Logically, the negative impacts of poor or inadequate access to healthcare are greater for those with high medical complexity, who require frequent interactions with a diverse range of services (James et al., 2019; Knox & Douglas, 2018). Such service need profiles are characteristic of traumatic and or other sudden onset injuries, most notably, acquired brain injury (ABI) and spinal cord injury (SCI) (DeJong et al., 2011; Guilcher et al., 2013; Knox & Douglas, 2018; Røe et al., 2019). In these complex rehabilitation populations, adequate access is critical to ameliorate high levels of disability (James et al., 2019). Unfortunately, people living with these lifelong disabilities regularly face major access barriers and high unmet needs (DeJong et al., 2011; Guilcher et al., 2013). High unmet needs can adversely affect quality of life, and lead to or exacerbate secondary complications, increasing the risk of further disability and premature death (James et al., 2019). Consequently, service infrastructure, and specifically, the accessibility of services, is a key consideration to avoid long-term functional complications, social adversity, and further disability (James et al., 2019; Knox & Douglas, 2018).

Access is a key indicator of health system performance (Levesque et al., 2013). Unfortunately, the origins of poor, or inadequate access, are remarkably complex—as are the potential barriers (Corscadden et al., 2017; Levesque et al., 2013). While there are unique disability-related differences due to the nature of impairments, common barriers across ABI and SCI are discernible. For example, barriers at the individual-level include: awareness (Guilcher et al., 2013; Ta'eed et al., 2013; Solovieva & Walls, 2014; McColl et al., 2008), funding situation (Guilcher et al., 2013; Røe et al., 2019; Solovieva & Walls, 2014), and point of care barriers like provider expertise (Knox & Douglas, 2018; McColl et al., 2008; Røe et al., 2019). Environmental-level barriers include physical accessibility and availability of services (Guilcher et al., 2013; Ta'eed et al., 2013; Solovieva & Walls, 2014; Kroll et al., 2006), and transportation-related obstacles (Darcy & Burke, 2018; Pyer & Tucker, 2017; Solovieva & Walls, 2014). Proximity of services has also been reported as a key challenge for accessing health services in the community (Guilcher et al., 2013; Solovieva & Walls, 2014). In the Australian context, the distribution of services across vast distances is another challenging planning issue (McGuirk & Argent, 2011; Weinhold & Gurtner, 2014).

In addition to proximity, the demand on available services can contribute to poor access, particularly if services cannot be accessed in a timely manner (James et al., 2019; Knox & Douglas, 2018). Decisions about and the supply and distribution of high-cost services, such as health and rehabilitation, often involve reconciling competing interests and demands (Knox & Douglas, 2018). This can lead to rationing and unevenness in terms of proximity and access (Knox & Douglas, 2018). Inadequate or inaccessible transportation can compound access issues, including lack of public transport infrastructure and costs (Lucas & Currie, 2012). Accessibility in terms of availability and affordability of services, including transport-related costs, rank high in access barriers for people with disability, requiring policy attention (World Health Organization, 2011). Alarmed by inequities in access, the WHO's (Gimigliano & Negrini, 2017) more recent report, *Rehabilitation 2030: a call for action*, has renewed attention on the uneven access and poor outcomes routinely experienced by people with lifelong disability. Citing the under-prioritisation of rehabilitation resources and infrastructure within the healthcare systems as a critical factor in poor access, unmet needs, and sub-optimal outcomes, WHO called on policy makers and clinicians to develop systemic solutions (Gimigliano & Negrini, 2017).

In Australia, consistent with the WHO's call to action (Gimigliano & Negrini, 2017), there have been calls for more detailed analyses of the health and service infrastructure, to improve access across the continuum of care for people with acquired disabilities (Middleton et al., 2014; Muenchberger et al., 2011). In Australia, where distance is a critical consideration for policy makers, the first step in response to this call to action is to map the current service infrastructure and assess what accessibility might resemble for specific populations. From that baseline, systemic strategies that improve access at a population-level may be contemplated. Using South-East Queensland, Australia as a starting point, this study aimed to: (a) describe accessibility, in terms of proximity to healthcare services commonly used by people with acquired disability; and (b) investigate whether potential service proximity was related to perceived service obstacles.

## 2 Methods

### 2.1 Study overview

The study comprised two parts. Briefly, we first described accessibility by conducting a spatial analysis to create maps of potential accessibility to health services in South-East Queensland. The analysis included Queensland statistical area level 2 (SA2; 2016 edition) location data (Australian Bureau of Statistics, 1270), along with locations from an ABI and SCI cohort (Legg et al., 2019), and health service data from Direct's National Health Service Directory (Healthdirect Australia, 2020). Travel times between each spatial location and the closest health service of interest were then modelled with Robust Gaussian Stochastic Process (Gu & Berger, 2016; Gu et al., 2018). Probabilistic predicted values were generated from the model and were used to construct service accessibility maps of South-East Queensland. Disability population data (Australian Bureau of Statistics, 2020a) were used to identify SA2s with relatively low service access but a high disability population. We focused on the top 10 ranked locations, with costs of non-independent travel via taxi also calculated for these top 10 ranked areas.

In second part of the study, we investigated whether service proximity was related to perceived service obstacles, using data from 63 people with ABI or SCI discharged from the specialist state-wide rehabilitation services, located in South-East Queensland. Obstacles to accessing service in relation to resource availability, transportation and finances were collected three-months after discharge. Travel times to the closest health service were computed for each individual and were included in models of service obstacle responses.

### 2.2 Describing accessibility

#### 2.2.1 Spatial data

The spatial dataset consisted of 485 datapoints: 320 SA2 centroid locations (2016 edition (Australian Bureau of Statistics, 1270)) and location data (residential address) on 165 people with ABI or SCI, who were participants in a longitudinal cohort study, recruited between March 2017 and March 2018 (Legg et al., 2019). Designed by the Australian Bureau of Statistics, SA2s are medium sized areas that aim to represent an interactive community, consisting of between 3000 and 25,000 people (Australian Bureau of Statistics, 1270). A list of the included SA2s is provided in Supplement 1. Participants in the longitudinal cohort study were recruited as inpatients, on a consecutive discharge basis from the specialist rehabilitation units at a tertiary hospital facility in South-East Queensland, Australia. Ethical approval was granted from the necessary Hospital (HREC/16/QPAH/684, SSA/16/QPAH/685) and University (2016/915) Human Research Ethics Committees. All participants, or their substitute decision makers, provided written informed consent before study involvement.

#### 2.2.2 Health and rehabilitation services

Medical specialist and allied health services most relevant to people with ABI or SCI were mapped. Medical specialist services included: rehabilitation medicine, neurology, neurosurgery, ophthalmology, orthopaedic surgery, plastic surgery, urology, and psychiatry. Allied health services included: psychology, speech pathology, dietetics, physiotherapy, exercise physiology, and occupational therapy. Hospitals and general practitioners were also mapped.

Except for rehabilitation medicine, the locations of health services were identified from Health Direct's National Health Service Directory—a national directory of health services, and practitioners who provide these services (Healthdirect Australia, 2020). The directory was accurate as of November 2019 and comprised approximately 300,000 health services. In Australia, Medicare, a universal health insurance scheme, entitles all citizens to access publicly funded hospital services and necessary medical services at no or minimal out-of-pocket cost (Foster & Fleming, 2008). Those with private health insurance also have access to the private hospital system and private allied health services (Foster & Fleming, 2008). Our analysis included both public and private services.

Health services could have been a practice, or a practitioner who works in a practice or in a hospital. The capacity of services listed in the directory (Healthdirect Australia, 2020) to deliver the service was not able to be distinguished. For example, a clinic may have had a neurosurgeon two days per week, or it could have had four neurosurgeons 24 h a day, seven days per week. Information regarding the capability of the medical specialist or allied health services to provide the sometimes highly specialised health and rehabilitation services required by people with ABI and SCI was not available. Physical accessibility information was also not available. We did not distinguish the level of hospital facility (e.g., tertiary facility) in our mapping.

Due to the inability to determine rehabilitation medicine services from other types of rehabilitation and restorative treatments in the National Health Service Directory, rehabilitation medicine locations were determined as per sites which had accredited training positions in rehabilitation medicine (Royal Australasian Colleague of Physicians, 2021). See Supplement 2 for a list of the included rehabilitation medicine services.

#### 2.2.3 Determination of travel times

Road network data were used to compute travel times, via motor vehicle, to the closest health provider. Road network data were derived from Geofabrik's OpenStreetMap (Geofabrik. OpenStreetMap Data, 2020), which accurately covers >90% of the Australian road network (Barrington-Leigh & Millard-Ball, 2017). Using R (R Core Team, 2020), the Open Source Routing Machine (Open Source Routing Machine, 2020) was used to establish the shortest travel time from each participant or SA2 location to the relevant health service. The route selected was the fastest based on motor vehicle travel via public roads, therefore, accounted for road types and variations in speed limits. The influence of traffic on travel times was not considered. Travel times were not specific to a particular time of day, or day of the week. A point on the road network closest to origins (locations) and destinations (health services) were used as beginning and endpoints. Travel time did not account for walking or parking, and therefore, provides a conservative estimation of the true travel time. In the instance where travel time to two health services were the same, the one with the shortest route was selected.

#### 2.2.4 Service accessibility mapping

The spatial data were modelled with Robust Gaussian Stochastic Process (Gu & Berger, 2016; Gu et al., 2018), to smoothly interpolate between the sparse time-to-service observations across the South-East Queensland region. Probabilistic predicted values were generated from the model at the centroids of a 100 by 400 hexagonal grid across the region of interest. Hexagons were approximately 1.5 km wide (East–West) and 1.9 km long (North–South). Predicted values were then used to construct the access maps, with travel times indicated in 15 min bounds. As the predictions are probabilistic, we report when the travel times exceed the chosen bounds with probability greater than 0.8. We used the R package RobustGaSP (Gu et al., 2020) to perform the inference for which code is available https://github.com/bonStats/healthcare-service-spatial.

The low-access versus high disability population ranking tables were created by averaging the grid of predictions within SA2 regions, to produce an average time-to-service access level per SA2 region. The metrics to rank regions were chosen as average time-to-service by (sub)population, identifying areas with relatively high (sub)populations and low-access to services. We used 2018 disability population data (Australian Bureau of Statistics, 2020a). In interpreting the results, we focused on the top 10 ranked locations in South-East Queensland, for each service of interest.

#### 2.2.5 Costs of travel

The cost of travel to health services via taxi was calculated, for the top 10 ranked low-access, high disability population areas. Costs were calculated from published rates (Department of Transport & Roads, 2020). In Queensland, taxi fares comprise several components: the distance travelled; the amount of waiting time during the journey (e.g., stopping at red lights, or in slow traffic); the time of day (i.e., a tariff); and are subject to a booking fee of AUD $1.50. The cost per distance travelled in South-East Queensland was $2.17/km. Because services were most likely to be accessed during business hours on weekdays, the 07:00 to 19:00 Monday to Friday tariff of $2.90 was used for all fare calculations. Fares were calculated based on the assumption that: (a) the journey was one-way, directly to the service; (b) no waiting time was incurred; and (c) the journey was exclusive of tolls. Based on these assumptions, the costs of travel are a conservative estimate only.

Taxi fares were calculated using Equation (1), where ‘*t*’ is the tariff (i.e., AUD $2.90), ‘*b*’ is the booking fare (i.e., AUD $1.50), ‘*d*’ is the distance from the SA2 of interest to the health service of interest, and ‘*k*’ is the cost per kilometre (i.e., AUD $2.17).

**Equation (1).** Cost of one-way travel via taxi, in Australian dollars.

Taxi fare (AUD $) = *t* + *b* + (*d* ⋅ *k*)

### 2.3 Examining perceived service obstacles

In the second part of the study, we investigated whether service proximity was related to perceived service obstacles. Sixty-three participants with an acquired disability were recruited between June 2019 to March 2020. Participants were inpatients at a tertiary hospital facility in South-East Queensland, Australia, recruited on a consecutive discharge basis from specialist rehabilitation (ABI or SCI, respectively). Participants were recruited as part of a larger study investigating access and wellbeing in these rehabilitation populations.

Participants were eligible for study inclusion if they: (a) had newly diagnosed ABI or SCI; (b) had capacity to provide informed consent, or if a substitute decision maker could provide consent on behalf of the participant, and (c) had the communication skills to complete a telephone survey or were able to complete the survey with the assistance from their substitute decision maker. All participants were also discharged to a private residence. Hospital (HREC/19/QMS/50271, SSA/19/QMS/50271) and University (2019/456) Human Research ethical clearance was granted for the project. All participants or their substitute decision makers provided written informed consent before study involvement.

Participants completed a telephone survey three-months after hospital discharge, where the Service Obstacles Scale (Marwitz & Kreutzer, 1996) was collected. Telephone surveys were administered by two researchers with clinical and research experience in ABI and SCI.

#### 2.3.1 Measures

Participant sociodemographic characteristics and disability related variables were retrieved from electronic hospital records. Sociodemographic variables included: age, gender, and marital status. Disability related variables included: disability and trauma type, and length of hospital stay. We also recorded whether participants received funding from the National Injury Insurance Scheme Queensland (NIISQ) (National Injury Insurance Scheme Queensland, 2021). Because some participants were discharged after the COVID-19 pandemic had been declared, we also made note of this, to account for it in our modelling.

Travel times to health and rehabilitation services were computed via the same methods used to describe accessibility. Participants are likely to access allied health services more regularly than medical specialist services (James et al., 2019). As such, we focused on participants mean travel time to allied health services—i.e., the average travel time across psychology, speech pathology, dietetics, physiotherapy, exercise physiology, and occupational therapy.

The Service Obstacles Scale was used to record the ease or difficulty participants experienced when accessing healthcare services in relation to service availability, transportation, and finances (Marwitz and Kreutzer, 1996). Participants rated the questions: ‘For injury related problems, there are very few resources in the community’ (item five); ‘Transportation is a major obstacle toward getting enough help’ (item two); and ‘Lack of money to pay for medical, rehabilitation, and injury related services is a major problem’ (item three) on a 7-point scale: 1 s*trongly disagree*, 2 *disagree*, 3 *slightly disagree*, 4 *neither agree nor disagree*, 5 *slightly agree*, 6 *agree*, and 7 *strongly agree*.

Transport independence is likely to be related to participants response to whether transportation is a major obstacle to accessing services (McGrail and Humphreys, 2009). The transportation item of the Mayo-Portland Adaptability Inventory-4 was used to capture participants’ level of transport independence (Malec and Lezak, 2008). Participants rated their independence as: 0 ‘Independent in all modes of transportation including independent ability to operate a personal motor vehicle’, 1 ‘Independent in all modes of transportation, but others have concerns about safety’, 2 ‘Requires a little assistance or supervision from others (5–24% of the time); cannot drive’, 3 ‘Requires moderate assistance or supervision from others (25–75% of the time); cannot drive’, and 4 ‘Requires extensive assistance or supervision from others (more than 75% of the time); cannot drive’.

#### 2.3.2 Data Analysis

Participant sociodemographic and disability related variables, and NIISQ funding support status were summarized as the median (interquartile range) or count (percent). Service obstacle responses were collapsed to ‘agree’ or ‘disagree’ before analysis. This was achieved by collapsing categories 1–3 (i.e., strongly disagree to slightly disagree) and categories 5–7 (i.e., slightly agree to strongly agree). Data were collapsed because there were several categories with few responses, for example, only two or three.

Participant responses (i.e., agree, disagree) to whether service availability, transportation, and finances were obstacles to accessing services were modelled using logistic regression. We did not include ‘neutral’ responses from transportation and finances obstacles due to low counts. For consistency we also modelled resource availability using logistic regression, including only the agree and disagree responses. However, because the neutral category accounted for 30% (n = 19) of responses to resource availability being an obstacle, we also fitted an ordinal regression model, to determine whether including the neutral ratings had any substantive effect on the results. When near identical, we reported the results from the logistic regression analysis. A separate model was fitted for each obstacle, with travel time (standardised, mean = 0) included as a predictor variable. All models also included rehabilitation type (levels: ABI, SCI) and whether (or not) participants three-month discharge timepoint fell after the COVID-19 pandemic had been declared as covariates. The transportation obstacle model adjusted for travel independence, and the finance obstacles model adjusted for whether participants had funding from the NIISQ.

Posterior estimates were based on 40,000 iterations (8 chains, 10,000 iterations, 50% burn-in). A Normal (mean = 0, SD = 2) prior distribution was used for the regression coefficients. Regression coefficients (on the logit scale) and odds ratios (OR) are reported as the mean and 95% credible interval (CrI). We also computed the posterior probability that a regression coefficient was greater than zero (Pr *β*>0), or less than zero (Pr *β*<0), depending on the direction of the effect. Posterior predictive checks were performed for all models. Models were fit in R (R Core Team, 2020) using the *brms* package (Bürkner, 2017).

## 3 Results

### 3.1 Describing Accessibility

#### 3.1.1 Distribution of Disability Population

**Fig. 1:**
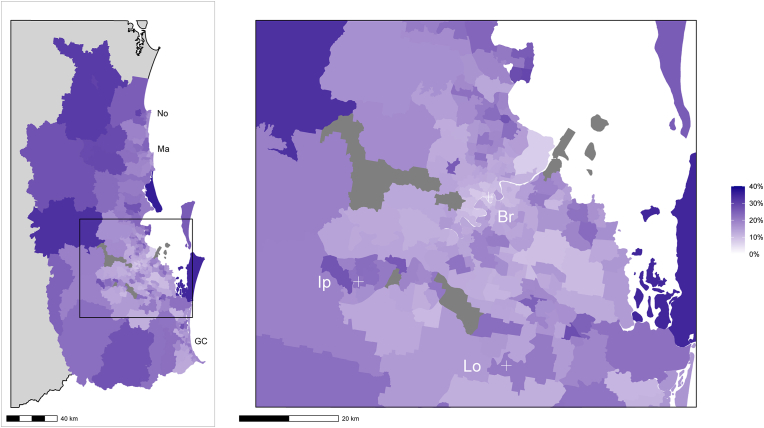
The proportion of people with disability residing in South-East Queensland statistical area level 2 (SA2) locations. Statistical areas are medium sized areas that aim to represent an interactive community, consisting of between 3000 and 25,000 people. Grey shade indicates an area with no data available. (For interpretation of the references to colour in this figure legend, the reader is referred to the Web version of this article.)

Figure 1 shows the proportion of people with disability residing in South-East Queensland, according to SA2 locations. There were higher proportions of people living with disability on the outskirts of the studied area, further away from the metropolitan region (i.e., greater Brisbane; Figure 1).

#### 3.1.2 Potential Access to Services

**Fig. 2:**
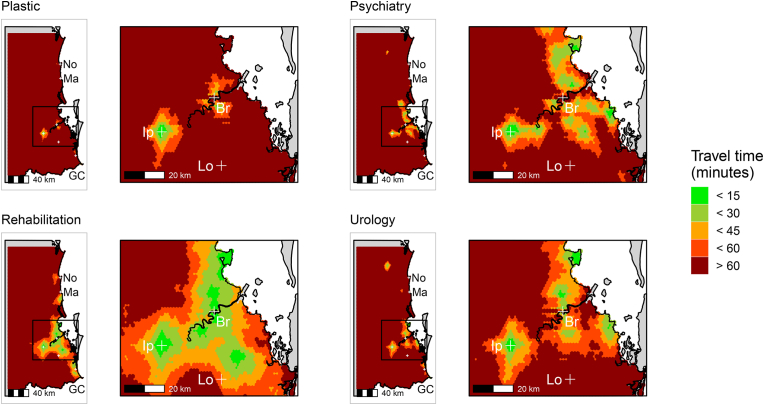
Maps of potential access in South-East Queensland to the medical specialist services of plastic surgery, psychiatry, rehabilitation medicine and urology. Br = Brisbane, Lo = Logan, Ip = Ipswich, No = Noosa, GC = Gold Coast, Ma = Maroochydore. (For interpretation of the references to colour in this figure legend, the reader is referred to the Web version of this article.)

**Fig. 3:**
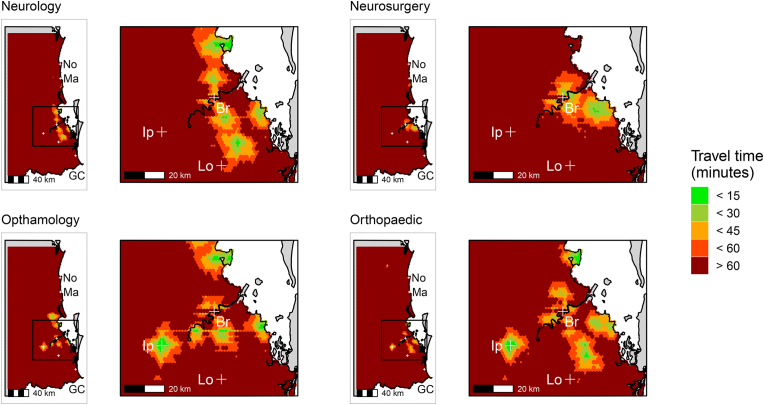
Maps of potential access in South-East Queensland to the medical specialist services of neurology, neurosurgery, ophthalmology, and orthopaedic surgery. Br = Brisbane, Lo = Logan, Ip = Ipswich, No = Noosa, GC = Gold Coast, Ma = Maroochydore. (For interpretation of the references to colour in this figure legend, the reader is referred to the Web version of this article.)

**Fig. 4:**
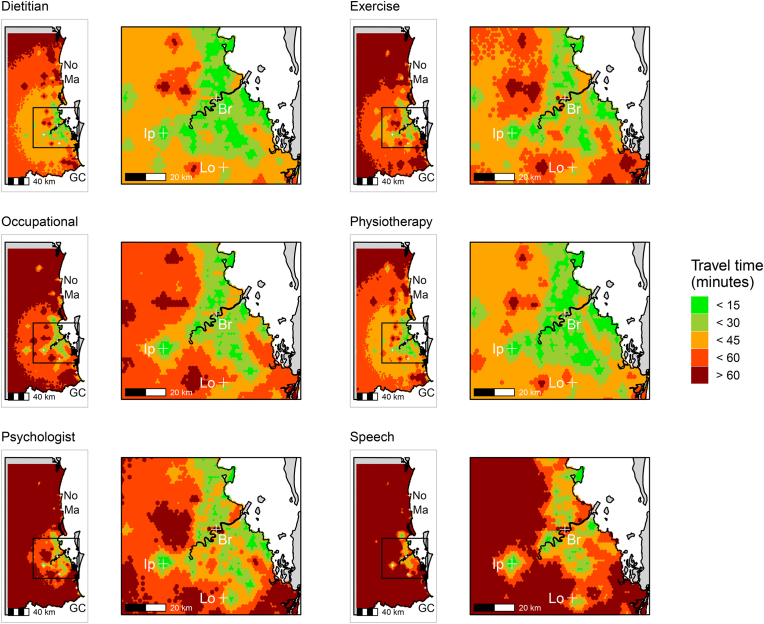
Maps of potential access in South-East Queensland to the allied health services of dietetics, exercise physiology, occupational therapy, physiotherapy, psychology, and speech pathology. Br = Brisbane, Lo = Logan, Ip = Ipswich, No = Noosa, GC = Gold Coast, Ma = Maroochydore. (For interpretation of the references to colour in this figure legend, the reader is referred to the Web version of this article.)

Maps of potential access to medical specialist services are shown in Fig. 2, Fig. 3, with maps of potential access to allied health services shown in Figure 4. As expected, across the studied region there was poorer potential accessibility to medical specialist services compared to allied health services, even in metropolitan locations (i.e., greater Brisbane). Of the medical specialities, there was greater potential access to rehabilitation medicine services than any other service (Fig. 2, Fig. 3). Maps of potential access to GPs and hospitals are shown in Appendix A, Appendix A, respectively. As expected, potential access to GPs was greatest of all the health services mapped.

While there was greater potential access to allied health services, there were still pockets of poor access in the metropolitan region, where travel times to allied health services exceeded 60 min (Figure 4). Across the studied region, potential access to dietetic and physiotherapy services was greatest, with the potential access to psychology and speech pathology services poorest (Figure 4). There was very poor access to speech pathology services even in the metropolitan region.

The top 10 ranked areas with low-service accessibility, but high-disability population for medical specialist services are shown in Table 1, with the top 10 ranked areas for allied health services shown in Table 2. Unsurprisingly, locations in the outer northern and western areas of the studied region, where the proportion of disability was highest and potential access lowest, featured regularly among the top 10 poor access areas. There was less variability in access potential across the top 10 ranked areas for allied health services compared to the top 10 ranked areas for medical specialist services. Potential cost of one-way travel via taxi to medical specialist services ranged from $8 to $311. The cost of one-way travel to allied health services ranged from $7 to $91.

**Table 1:** The top 10 ranked statistical area level 2 (SA2) regions with low-service accessibility but high-disability population for medical specialist services. The mean travel time, general and disability population data, and potential one-way travel cost via taxi to the service of interest is reported for each SA2.

Rank	Statistical area level 2 region	Mean travel time (min)	General population	Disability population	Proportion of disability	Index	Travel cost ($AUD)
Neurology							
1	Maroochydore - Kuluin	225	20533	4965	0.24	1	38
2	Gympie Region	142	19673	6109	0.31	0.775	160
3	Noosa Hinterland	143	23358	5891	0.25	0.752	108
4	Gympie - North	169	14743	4385	0.30	0.663	160
5	Bribie Island	95	19268	6974	0.36	0.589	126
6	Buderim - North	170	17692	3530	0.20	0.536	41
7	Upper Coomera - Willow Vale	79	34878	7483	0.21	0.531	56
8	Maryborough Region - South	170	8787	3325	0.38	0.505	311
9	Nanango	131	9767	4007	0.41	0.468	79
10	Marcoola - Mudjimba	172	11905	2880	0.24	0.444	39
Neurosurgery							
1	Surfers Paradise	606	26486	4691	0.18	1	161
2	Robina	443	25489	3767	0.15	0.587	174
3	Southport - South	549	18545	2897	0.16	0.560	155
4	Mermaid Waters	547	12782	2650	0.21	0.510	172
5	Carrara	501	13061	2840	0.22	0.501	157
6	Nerang - Mount Nathan	256	21676	4822	0.22	0.435	156
7	Mermaid Beach - Broadbeach	639	13134	1906	0.15	0.429	173
8	Ashmore	455	12481	2645	0.21	0.424	151
9	Burleigh Waters	377	14799	3175	0.21	0.421	186
10	Gympie Region	180	19673	6109	0.31	0.387	210
Ophthalmology							
1	Gympie Region	124	19673	6109	0.31	1	138
2	Gympie - North	165	14743	4385	0.30	0.9557	138
3	Upper Coomera - Willow Vale	82	34878	7483	0.21	0.8135	56
4	Noosa Hinterland	100	23358	5891	0.25	0.7780	50
5	Bribie Island	75	19268	6974	0.36	0.6961	91
6	Nambour	115	21059	4558	0.22	0.6960	42
7	Maryborough Region - South	146	8787	3325	0.38	0.6434	84
8	Nerang - Mount Nathan	96	21676	4822	0.22	0.6145	33
9	Nanango	112	9767	4007	0.41	0.5935	76
10	Maroochydore - Kuluin	83	20533	4965	0.24	0.5451	10
Orthopaedic surgery							
1	Gympie Region	111	19673	6109	0.31	1	25
2	Bribie Island	94	19268	6974	0.36	0.963	137
3	Noosa Hinterland	107	23358	5891	0.25	0.926	50
4	Caboolture	72	29132	7850	0.27	0.831	70
5	Maryborough Region - South	157	8787	3325	0.38	0.765	160
6	Surfers Paradise	104	26486	4691	0.18	0.713	15
7	Upper Coomera - Willow Vale	63	34878	7483	0.21	0.690	32
8	Nanango	116	9767	4007	0.41	0.685	76
9	Maroochydore - Kuluin	87	20533	4965	0.24	0.633	8
10	Cooloola	137	6487	3111	0.48	0.624	112
Plastic surgery							
1	Maroochydore - Kuluin	455	20533	4965	0.24	1	87
2	Buderim - North	345	17692	3530	0.20	0.539	91
3	Buderim - South	314	17347	3183	0.18	0.441	115
4	Bribie Island	139	19268	6974	0.36	0.429	175
5	Caloundra - West	223	23775	4272	0.18	0.421	131
6	Gympie Region	151	19673	6109	0.31	0.409	138
7	Marcoola - Mudjimba	303	11905	2880	0.24	0.386	76
8	Mooloolaba - Alexandra Headland	434	12408	1933	0.16	0.371	97
9	Nambour	177	21059	4558	0.22	0.357	83
10	Caboolture	94	29132	7850	0.27	0.327	108
Psychiatry							
1	Gympie Region	93	19673	6109	0.31	1	25
2	Noosa Hinterland	93	23358	5891	0.25	0.969	51
3	Bribie Island	75	19268	6974	0.36	0.927	94
4	Nambour	100	21059	4558	0.22	0.802	37
5	Nerang - Mount Nathan	91	21676	4822	0.22	0.772	34
6	Maryborough Region - South	129	8787	3325	0.38	0.757	160
7	Maroochydore - Kuluin	83	20533	4965	0.24	0.729	9
8	Upper Coomera - Willow Vale	55	34878	7483	0.21	0.727	32
9	Nanango	98	9767	4007	0.41	0.693	283
10	Coolum Beach	139	15791	2676	0.17	0.656	48
Rehabilitation Medicine							
1	Bribie Island	55	19268	6974	0.36	1	137
2	Caboolture	43	29132	7850	0.27	0.869	70
3	Gympie Region	52	19673	6109	0.31	0.829	108
4	Upper Coomera - Willow Vale	38	34878	7483	0.21	0.732	56
5	Noosa Hinterland	46	23358	5891	0.25	0.697	49
6	Gympie - North	61	14743	4385	0.30	0.696	108
7	Lockyer Valley - East	48	21105	5181	0.25	0.640	107
8	Maroochydore - Kuluin	45	20533	4965	0.24	0.581	38
9	Nambour	49	21059	4558	0.22	0.580	67
10	Beaudesert	52	14788	3983	0.27	0.541	158
Urology							
1	Bribie Island	88	19268	6974	0.36	1	137
2	Noosa Hinterland	96	23358	5891	0.25	0.9210	106
3	Upper Coomera - Willow Vale	63	34878	7483	0.21	0.7599	56
4	Caboolture	57	29132	7850	0.27	0.7286	70
5	Gympie Region	71	19673	6109	0.31	0.7015	25
6	Nanango	88	9767	4007	0.41	0.5694	283
7	Lockyer Valley - East	67	21105	5181	0.25	0.5666	100
8	Tewantin	109	10589	3165	0.30	0.5589	88
9	Beaudesert	83	14788	3983	0.27	0.5373	167
10	Maryborough Region - South	100	8787	3325	0.38	0.5370	160

Note. Statistical area level 2 (SA2) regions were ranked according to the (scaled) index, which was calculated as the average time-to-service by disability population. Areas with relatively high disability populations and low access to services were highly ranked. AUD = Australian dollars.

**Table 2:** The top 10 ranked statistical area level 2 (SA2) regions with low-service accessibility but high-disability population for allied health services. The mean travel time, general and disability population data, and potential one-way travel cost via taxi to the service of interest is reported for each SA2.

Rank	Statistical area level 2 region	Mean travel time (min)	Disability pop.	General pop.	Prop. of disability	Index	Travel cost ($)
Dietetics							
1	Upper Coomera - Willow Vale	25	34878	7483	0.21	1	35
2	Gympie Region	28	19673	6109	0.31	0.9180	24
3	Noosa Hinterland	29	23358	5891	0.25	0.9178	46
4	Maroochydore - Kuluin	33	20533	4965	0.24	0.8978	11
5	Bribie Island	23	19268	6974	0.36	0.8671	39
6	Nerang - Mount Nathan	28	21676	4822	0.22	0.7341	19
7	Buderim - North	33	17692	3530	0.20	0.6352	11
8	Maryborough Region - South	33	8787	3325	0.38	0.5992	28
9	Nanango	27	9767	4007	0.41	0.5833	29
10	Caboolture	14	29132	7850	0.27	0.5787	12
Exercise physiology							
1	Upper Coomera - Willow Vale	33	34878	7483	0.21	1	32
2	Noosa Hinterland	38	23358	5891	0.25	0.920	49
3	Gympie Region	37	19673	6109	0.31	0.913	24
4	Bribie Island	29	19268	6974	0.36	0.827	39
5	Nerang - Mount Nathan	33	21676	4822	0.22	0.651	15
6	Maryborough Region - South	45	8787	3325	0.38	0.608	28
7	Nanango	36	9767	4007	0.41	0.587	24
8	Carrara	49	13061	2840	0.22	0.566	16
9	Surfers Paradise	28	26486	4691	0.18	0.545	7
10	Buderim - North	38	17692	3530	0.20	0.541	11
Occupational therapy							
1	Bribie Island	37	19268	6974	0.36	1	91
2	Noosa Hinterland	41	23358	5891	0.25	0.914	49
3	Gympie Region	38	19673	6109	0.31	0.897	24
4	Upper Coomera - Willow Vale	30	34878	7483	0.21	0.858	32
5	Nerang - Mount Nathan	44	21676	4822	0.22	0.814	37
6	Coolum Beach	75	15791	2676	0.17	0.769	45
7	Maroochydore - Kuluin	38	20533	4965	0.24	0.730	11
8	Surfers Paradise	34	26486	4691	0.18	0.619	10
9	Maryborough Region - South	47	8787	3325	0.38	0.598	84
10	Marcoola - Mudjimba	54	11905	2880	0.24	0.593	22
Physiotherapy							
1	Noosa Hinterland	30	23358	5891	0.25	1	48
2	Gympie Region	28	19673	6109	0.31	0.975	20
3	Upper Coomera - Willow Vale	22	34878	7483	0.21	0.922	24
4	Bribie Island	22	19268	6974	0.36	0.893	33
5	Maroochydore - Kuluin	30	20533	4965	0.24	0.856	9
6	Robina	35	25489	3767	0.15	0.757	10
7	Surfers Paradise	25	26486	4691	0.18	0.662	7
8	Maryborough Region - South	34	8787	3325	0.38	0.653	83
9	Nerang - Mount Nathan	23	21676	4822	0.22	0.647	16
10	Buderim - North	32	17692	3530	0.20	0.645	10
Psychology							
1	Gympie Region	50	19673	6109	0.31	1	24
2	Maroochydore - Kuluin	59	20533	4965	0.24	0.947	8
3	Noosa Hinterland	49	23358	5891	0.25	0.932	31
4	Upper Coomera - Willow Vale	38	34878	7483	0.21	0.915	33
5	Bribie Island	35	19268	6974	0.36	0.802	39
6	Nerang - Mount Nathan	47	21676	4822	0.22	0.739	16
7	Maryborough Region - South	65	8787	3325	0.38	0.708	84
8	Buderim - North	60	17692	3530	0.20	0.686	10
9	Robina	55	25489	3767	0.15	0.675	10
10	Carrara	71	13061	2840	0.22	0.652	12
Speech pathology							
1	Gympie Region	65	19673	6109	0.31	1	24
2	Noosa Hinterland	64	23358	5891	0.25	0.940	49
3	Surfers Paradise	79	26486	4691	0.18	0.926	14
4	Bribie Island	51	19268	6974	0.36	0.882	91
5	Maroochydore - Kuluin	68	20533	4965	0.24	0.849	9
6	Upper Coomera - Willow Vale	44	34878	7483	0.21	0.831	38
7	Coolum Beach	115	15791	2676	0.17	0.767	45
8	Maryborough Region - South	85	8787	3325	0.38	0.708	87
9	Nerang - Mount Nathan	56	21676	4822	0.22	0.678	24
10	Nanango	64	9767	4007	0.41	0.645	24

Note. Statistical area level 2 (SA2) regions were ranked according to the (scaled) index, which was calculated as the average time-to-service by disability population. Areas with relatively high disability populations and low access to services were highly ranked. AUD = Australian dollars.

### 3.2 Examining Perceived Service Obstacles

Participant sociodemographic characteristics, disability related variables and service obstacles responses are summarised in Table 3. Participants median age was 48 years, and they were generally male (71%), married or in a de facto relationship (64%). There was an even representation of participants across the acquired disabilities of ABI and SCI (Table 3). Most participants (87%) were not able to drive at three-months after discharge.

**Table 3:** Participant sociodemographic and disability related variables and service obstacle responses.

Variable	N = 63
Age, median (interquartile range) years	48 (40–59)
Gender—male, n (%)	45 (71%)
Marital status, n (%)	
Married/de facto	39/61 (64%)
Divorced/separated	8/61 (13%)
Never married	14/61 (23%)
Geography, n (%)	
Major city of Australia	43 (68%)
Inner regional Australia	15 (24%)
Outer regional and remote Australia	5 (8%)
Primary disability, n (%)	
Brain injury	32 (51%)
Spinal cord injury	31 (49%)
Trauma type—traumatic injury, n (%)	33 (52%)
Length of hospital stay, median (interquartile range) days	103 (39–117)
National injury insurance scheme funded—Yes, n (%)	12 (19%)
Travel independence, n (%)	
Independent in all modes of transportation including independent ability to operate a personal motor vehicle	8 (13%)
Independent in all modes of transportation, but others have concerns about safety	2 (3%)
Requires a little assistance or supervision from others 5–24% of the time; cannot drive	21 (33%)
Requires moderate assistance or supervision from others 25–75% of the time	18 (29%)
Requires extensive assistance or supervision from others more than 75% of the time; cannot drive	14 (22%)
Service Obstacles Scale	
For injury related problems, there are very few resources in the community, n (%)	
Disagree	26 (41%)
Neutral	19 (30%)
Agree	18 (29%)
Transportation is a major obstacle toward getting enough help, n (%)	
Disagree	33 (52%)
Neutral	2 (3%)
Agree	28 (44%)
Lack of money to pay for medical, rehabilitation, and injury related services is a major problem, n (%)	
Disagree	42 (67%)
Neutral	4 (6%)
Agree	17 (27%)

Note. Age and length of stay are reported as the median and interquartile range, with all other variables are reported as count and percent. Percentages may not sum exactly to 100 due to rounding.

Forty-seven participants (25%) lived within the area mapped in the first part of the study, where we described accessibility. Of these 47 participants, two lived in the top 10 ranked low-access, high disability areas—one participant for two medical specialist services, and another participant for all allied health and most medical specialist services.

About one-third of participants (n = 18; 29%) agreed that resource availability was an obstacle to accessing services, with two-in-five (n = 28; 44%) and one-in-four (n = 17; 27%) participants indicating that transportation and finances were an obstacle to accessing services (Table 3). Participants generally lived in a major city (68%). The median (interquartile range) average travel time to allied health services was 7.12 min (3.02–8.67), with a range of 0.79–49.60 min.

**Fig. 5:**
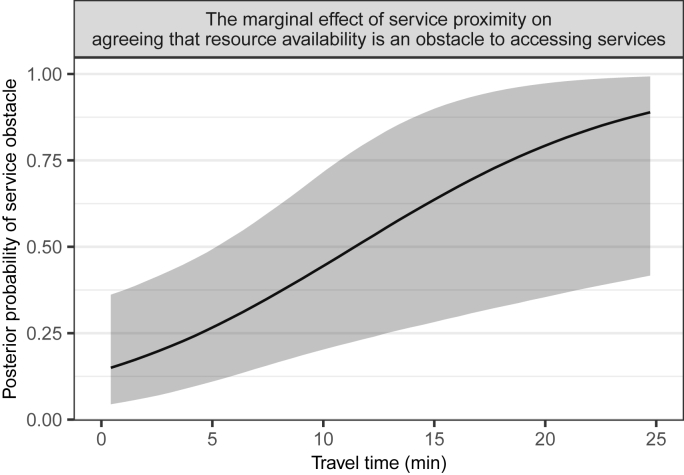
The marginal effect of mean allied health service proximity, in terms of travel times, on resource availability being an obstacle to accessing services. Black solid line indicates the posterior mean, and the grey ribbon indicates the 95% credible interval. Longer travel times were associated with higher odds of agreeing that for injury related problems there were few healthcare services in the community.

There was evidence that longer travel times to allied health services were associated with resource availability obstacles (*β* = 1.28, 95% CrI = 0.33, 2.39; Pr *β*>0 = 0.998). Longer travel times were associated with 260% higher odds of agreeing that for injury related problems there were few healthcare services in the community (OR = 3.60, 95% CrI = 1.39, 10.91; Fig. 5). We did not find any evidence that longer travel times to allied health services were associated with agreeing that transportation (*β* = 0.12, 95% CrI = −0.49, 0.72; Pr *β*>0 = 0.660) or finances (*β* = −0.28, 95% CrI = −0.90, 0.31; Pr *β*<0 = 0.824) were an obstacle to accessing services.

## 4 Discussion

This study (a) described the accessibility of healthcare services in South-East Queensland, in terms of potential proximity to services commonly used by people with an acquired disability; and (b) investigated whether potential service proximity was related to perceived service obstacles in a cohort of participants with ABI and SCI recently discharged to the community. There was considerable variability in access potential, which in general, was greater for allied health services (Fig. 4) compared to medical specialist services (Fig. 2, Fig. 3). We highlighted low-access high disability population areas, which were generally to the north and west of greater Brisbane (Table 1, Table 2). Poorer potential access to allied health services, in terms of longer travel times, was associated with resource availability being rated as an obstacle to accessing services (Fig. 5). We did not find any evidence that proximity to allied health services was associated with transportation or finance obstacles. However, further investigation is needed to confirm these findings. Policy makers should be concerned with the inequalities in potential access experienced by people living with acquired disabilities in these hotspots of poor access.

Irrespective of the geographical location, a population can have poor access (McGuirk & Argent, 2011). We found evidence of this in the current analysis, where even in urban areas, potential access was poor (e.g., speech pathology; Fig. 4). There was large variability in access potential across the services mapped; however, the significance of this variability is difficult to interpret, because an even distribution of services does not necessarily imply sufficiency (Morell et al., 2017). Using general disability population data as an indicator of potential service demand, we were able to highlight several low-access, high disability population areas which could be considered as hotspots of poor access. These hotspots were generally to the north and west of greater Brisbane (Table 1, Table 2). As expected, there was geographical disadvantage for outer suburbia locations in terms of potential access to medical specialist services (Fig. 2, Fig. 3) (Keeves et al., 2021). Although the urban centralisation of medical specialist services is necessary (Lawrence & Fudge, 2009), it has the potential to be problematic for suburban people with complex rehabilitation needs, who require regular and timely access to these services (Solovieva & Walls, 2014). Our analysis shows that demands on health services from general disability populations may be greater in outer suburban areas (Table 2, Table 3). As such, people with acquired disability discharged to low-access, high disability population areas may be at increased risk of experiencing unmet need, and therefore, further disability and premature death (James et al., 2019; Solovieva & Walls, 2014).

Housing affordability is important for service and infrastructure planning (Okkola & Brunelle, 2018). In the examination of perceived service obstacles, 27% (n = 17) of participants indicated that finances were a major problem to accessing healthcare services (Table 3). It is reasonable to suggest that these individuals may also experience financial hardship in other areas of life, such as housing (Okkola & Brunelle, 2018). The dispersion of people with disability into outer suburbia in search of more affordable housing is a major planning issue (Okkola & Brunelle, 2018; Willing & Pojani, 2017). Unaffordable housing in urban areas could explain our observations of higher proportions of people with general disabilities living in the outer regions of the studied area (Fig. 1). Despite needing regular and timely access to services, people may simply live where they can afford. Equally, suburban living may be a choice and a preference for ‘peace and quiet’ and slower paced living, rather than being due to housing unaffordability in urban areas (Willing & Pojani, 2017).

A lack of housing affordability can lead to regular changes in housing, referred to as residential mobility (Baker et al., 2016). Increased residential mobility has the potential to negatively impact a person's continuity of healthcare, which could be particularly detrimental during the early phases of rehabilitation and in populations with complex needs (James et al., 2019; Knox & Douglas, 2018). Accordingly, the planning of affordable housing is a key consideration for policy makers to ensure equitable healthcare access, especially when considering population shifts. Unsurprisingly, population growth adds further pressure to housing affordability, which in the current climate, is highly relevant for South-East Queensland (Willing & Pojani, 2017). Even before the COVID-19 pandemic, Queensland had seen the highest yearly population growth in Australia at 1.1% (Australian Bureau of Statistics, 2020b), with population growth projections of up to 2.1% by 2041 (Queensland Government Statistician's Office, 2021). It is imperative to people with acquired disability that affordable housing and accessible healthcare services are prioritised by policy makers.

While housing affordability improves further away from urban areas, transportation disadvantage worsens (Haffner & Hulse, 2021). Access to transport is a key facilitator of timely access to healthcare (McGrail & Humphreys, 2009). When examining perceived service obstacles, and as expected (Kolakowsky-Hayner et al., 2000; Kroll et al., 2006; Solovieva & Walls, 2014), a considerable proportion (n = 28; 44%) of participants agreed that transportation was a major obstacle to accessing services. Although this result could be explained by participants inability to drive three-months after hospital discharge, it is worth acknowledging that public transport is usually limited, or infrequent, further away from urban areas—although this can also occur in major Australian cities (McGrail & Humphreys, 2009). Public transport anxiety is commonly reported by people with acquired disability (Pyer & Tucker, 2017). Poor infrastructure (e.g., ramps, buzzers) and regulations were found to exacerbate the level of transport anxiety experienced (Pyer & Tucker, 2017). For example, people with SCI have reported space conflict with parents pushing prams (Pyer & Tucker, 2017). Should the policy be ‘first come, first served’ or should the wheelchair user have priority? (Velho, 2019). Regardless, increased anxiety in relation to transport has the potential to negatively affect service use, or a person's willingness to travel via public transport in order to access services, and therefore, could lead to unmet needs (Pyer & Tucker, 2017; Velho, 2019). Improving accessibility to public transport, and space regulations, could be an effective policy for governments to improve healthcare accessibility, from within transportation systems (Brown et al., 2019).

People accessing services via private transport may require a family member, friend or carer to drive them (Jan et al., 2012). For people with SCI, vehicle modifications, regardless of whether the person is the driver or the passenger, are generally required (Darcy & Burke, 2018). Both transportation via carers (Jan et al., 2012) and vehicle modifications (Darcy & Burke, 2018) can incur significant financial cost, exacerbating financial hardship. While non-independent travel (e.g., taxis) are another transportation option, this mode of transport can be costly, as highlighted in our analysis (Table 2, Table 3). While Australia has a Taxi Subsidy Scheme for people with severe disabilities, which reimburses some of the taxi fare, up to a maximum of AUD $30, users still incur some out-of-pocket expense, which could prove significant, particularly for those individuals who require frequent service use, or who travel long distances (Queensland Government, 2022). Telehealth may alleviate some of the systematic barriers and the need for transportation; however, telehealth requires a change in service delivery, discipline specific training, and innovation (Cole et al., 2019; van de Pol et al., 2016). Telehealth may introduce other disadvantage, particularly in terms of internet access and connectivity, which is often poorer further away from urban areas (Kaplan & Litewka, 2008). Receiving services via telehealth may also not suit everyone.

Our analysis is a best-case scenario of potential access to healthcare for people acquired ABI or SCI. While the current study provides an indication of potential access to services, it does not provide any information on service quality, service capacity, the suitability for people with complex rehabilitation needs, or the links between primary, acute, and rehabilitative services. Health care quality is an important policy concern, as deficiencies can contribute to shortages of sufficient healthcare, particularly in non-urban areas (Weinhold & Gurtner, 2014). User preference is not considered in our analysis, but may be a strong motivator for travelling further to access healthcare, and is linked with satisfaction (Liu et al., 2018). Interestingly, lower user satisfaction has been reported in more economically prosperous areas, which may be due to people providing higher ratings of satisfaction in areas where service availability is lower (Liu et al., 2018).

The physical accessibility to the mapped services, an important consideration for wheelchair or mobility users, is not known. It is not possible to distinguish between public and private services, which may limit peoples access to services as not everyone is willing or has the capacity to pay for services. Results from the examination of perceived service obstacles are not generalisable due to the small sample size, and therefore, are specific to the 63 people for which data were available. Our analysis does not consider access to services in outer regional or remote areas of Queensland. Travel time in these areas has different magnitude and variability to metropolitan regions, and so can be inappropriate to model jointly. Moreover, our attention to SA2 data leads to outer areas being very large and difficult to model accurately. We also note that travel time in outer regional or remote areas may not reflect actual travel patterns with patients flying or moving temporarily for ongoing treatment. We used the most current health service data available at the time of analysis. However, participant location data used in the describing accessibility analysis came from an investigation conducted before 2019, which may be a limitation of the current study.

Some services may have significant wait times. The impact of wait times on potential access shoulder be considered by future studies. Future studies should also investigate the relationship between potential and realised access for people with acquired disability; determine people's willingness to travel to services due to preference; and examine the association between service use satisfaction and patterns of realised access. There is also a need for investigations that focus on the preferences of people with disabilities with respect to accessing healthcare and associated changes to improve the health service (i.e., discrete choice experiments).

## 5 Conclusion

There was considerable variability in access potential in South-East Queensland, which was generally greater for allied health services than medical specialist services. We have identified low-access high disability population areas, which were typically located to the north and west of the studied area. Policy makers should be concerned with these hotspots of poor access. In a small sample of people with acquired disability we found evidence that service proximity was associated with resource availability being an obstacle to accessing services. However, future investigation is needed to confirm these findings. When considering the limitations of the analysis such as service quality, capacity, physical accessibility, and specialisation, these findings are an underestimation of the true potential for accessibility hardship. Our findings should be considered in the planning of future infrastructure, to mitigate inequities in service accessibility, with a particularly focus on the hotspots of poor access identified. Without adequate health service infrastructure, and transportation in poor access areas, particularly in the context of population growth, people with disability living in South-East Queensland will continue to face unnecessary hardship, in terms of service accessibility, increasing the risk of unmet need, further disability and ultimately, premature death.

## Author CRediT statement

**David N Borg:** Conceptualization; Data curation; Formal analysis; Investigation; Methodology; Visualization; Original draft, review and editing. **Joshua J Bon:** Conceptualization; Data curation; Formal analysis; Methodology; Visualization; Original draft, review and editing. **Michele M Foster:** Conceptualization; Funding acquisition; Investigation; Methodology; Supervision; Original draft, review and editing. **Ali Lakhani:** Formal analysis; Methodology; Original draft, review and editing. **Melissa Kendall:** Conceptualization; Funding acquisition; Investigation; Methodology; Supervision; Original draft, review and editing. **Timothy Geraghty:** Conceptualization; Funding acquisition; Methodology; Supervision; Original draft, review and editing.

## Ethical statement

Ethical approval was granted from the necessary Hospital (HREC/16/QPAH/684, SSA/16/QPAH/685; HREC/19/QMS/50271, SSA/19/QMS/50271) and University (2016/915; 2019/456) Human Research Ethics Committees. All participants provided informed consent before study involvement.

## Funding

This work was supported by funding from the 10.13039/100010398Metro South Health Research Support Scheme. The funder had no role in the study design, collection of data, data analysis or interpretation of the results, or the decision to publish this work.

## Declaration of competing interest

None.

## Data Availability

The data and R code for the spatial analysis are available at https://github.com/bonStats/healthcare-service-spatial.
