# Supplementary material for: Healthcare services for people with acquired disability in South-East Queensland, Australia: Assessing potential proximity and its association with service obstacles
Source: SSM Popul Health. 2022 Aug 17;19:101209. doi: 10.1016/j.ssmph.2022.101209 (PMC9424535; doi:10.1016/j.ssmph.2022.101209)
Supplement: Multimedia component 1 [file mmc1.docx]

**Supplement 1.** List of mapped SA2s.

| **SA2 code** | **SA2 name** |
| --- | --- |
| 301011001 | Alexandra Hills |
| 301011002 | Belmont - Gumdale |
| 301011003 | Birkdale |
| 301011004 | Capalaba |
| 301011005 | Thorneside |
| 301011006 | Wellington Point |
| 301021007 | Cleveland |
| 301021008 | Ormiston |
| 301021009 | Redland Bay |
| 301021011 | Sheldon - Mount Cotton |
| 301021012 | Thornlands |
| 301021013 | Victoria Point |
| 301021527 | Redland Islands |
| 301031014 | Brisbane Port - Lytton |
| 301031015 | Manly - Lota |
| 301031016 | Manly West |
| 301031017 | Murarrie |
| 301031018 | Tingalpa |
| 301031019 | Wakerley |
| 301031020 | Wynnum |
| 301031021 | Wynnum West - Hemmant |
| 302011022 | Bald Hills |
| 302011023 | Bridgeman Downs |
| 302011024 | Carseldine |
| 302011025 | Everton Park |
| 302011026 | McDowall |
| 302021027 | Aspley |
| 302021028 | Chermside |
| 302021029 | Chermside West |
| 302021030 | Geebung |
| 302021031 | Kedron - Gordon Park |
| 302021032 | Stafford |
| 302021033 | Stafford Heights |
| 302021034 | Wavell Heights |
| 302031035 | Boondall |
| 302031036 | Brisbane Airport |
| 302031037 | Eagle Farm - Pinkenba |
| 302031038 | Northgate - Virginia |
| 302031039 | Nudgee - Banyo |
| 302031040 | Nundah |
| 302041041 | Bracken Ridge |
| 302041042 | Brighton (Qld) |
| 302041043 | Deagon |
| 302041044 | Sandgate - Shorncliffe |
| 302041045 | Taigum - Fitzgibbon |
| 302041046 | Zillmere |
| 303011047 | Camp Hill |
| 303011048 | Cannon Hill |
| 303011049 | Carina |
| 303011050 | Carina Heights |
| 303011051 | Carindale |
| 303021052 | Annerley |
| 303021053 | Coorparoo |
| 303021054 | Fairfield - Dutton Park |
| 303021055 | Greenslopes |
| 303021056 | Holland Park |
| 303021057 | Holland Park West |
| 303021058 | Woolloongabba |
| 303021059 | Yeronga |
| 303031060 | Eight Mile Plains |
| 303031061 | Macgregor (Qld) |
| 303031062 | Mansfield (Qld) |
| 303031063 | Mount Gravatt |
| 303031064 | Rochedale - Burbank |
| 303031065 | Upper Mount Gravatt |
| 303031066 | Wishart |
| 303041067 | Coopers Plains |
| 303041068 | Moorooka |
| 303041069 | Robertson |
| 303041070 | Salisbury - Nathan |
| 303041071 | Tarragindi |
| 303051072 | Algester |
| 303051073 | Calamvale - Stretton |
| 303051074 | Pallara - Willawong |
| 303051075 | Parkinson - Drewvale |
| 303051076 | Rocklea - Acacia Ridge |
| 303061077 | Kuraby |
| 303061078 | Runcorn |
| 303061079 | Sunnybank |
| 303061080 | Sunnybank Hills |
| 304011081 | Jindalee - Mount Ommaney |
| 304011082 | Middle Park - Jamboree Heights |
| 304011083 | Riverhills |
| 304011084 | Seventeen Mile Rocks - Sinnamon Park |
| 304011085 | Westlake |
| 304021086 | Bellbowrie - Moggill |
| 304021087 | Brookfield - Kenmore Hills |
| 304021088 | Chapel Hill |
| 304021089 | Fig Tree Pocket |
| 304021090 | Kenmore |
| 304021091 | Pinjarra Hills - Pullenvale |
| 304031092 | Chelmer - Graceville |
| 304031093 | Corinda |
| 304031094 | Indooroopilly |
| 304031095 | Sherwood |
| 304031096 | St Lucia |
| 304031097 | Taringa |
| 304041098 | Enoggera |
| 304041099 | Enoggera Reservoir |
| 304041100 | Keperra |
| 304041101 | Mitchelton |
| 304041102 | Mount Coot-tha |
| 304041103 | The Gap |
| 304041104 | Upper Kedron - Ferny Grove |
| 305011105 | Brisbane City |
| 305011106 | Fortitude Valley |
| 305011107 | Highgate Hill |
| 305011108 | Kangaroo Point |
| 305011109 | New Farm |
| 305011110 | South Brisbane |
| 305011111 | Spring Hill |
| 305011112 | West End |
| 305021113 | Balmoral |
| 305021114 | Bulimba |
| 305021115 | East Brisbane |
| 305021116 | Hawthorne |
| 305021117 | Morningside - Seven Hills |
| 305021118 | Norman Park |
| 305031119 | Albion |
| 305031120 | Alderley |
| 305031121 | Ascot |
| 305031122 | Clayfield |
| 305031123 | Grange |
| 305031124 | Hamilton (Qld) |
| 305031125 | Hendra |
| 305031126 | Kelvin Grove - Herston |
| 305031127 | Newmarket |
| 305031128 | Newstead - Bowen Hills |
| 305031129 | Wilston |
| 305031130 | Windsor |
| 305031131 | Wooloowin - Lutwyche |
| 305041132 | Ashgrove |
| 305041133 | Auchenflower |
| 305041134 | Bardon |
| 305041135 | Paddington - Milton |
| 305041136 | Red Hill (Qld) |
| 305041137 | Toowong |
| 309011224 | Broadbeach Waters |
| 309011225 | Burleigh Heads |
| 309011226 | Burleigh Waters |
| 309011227 | Mermaid Beach - Broadbeach |
| 309011228 | Mermaid Waters |
| 309011229 | Miami |
| 309021230 | Coolangatta |
| 309021231 | Currumbin - Tugun |
| 309021232 | Currumbin Waters |
| 309021233 | Elanora |
| 309021234 | Palm Beach |
| 309031235 | Arundel |
| 309031236 | Biggera Waters |
| 309031237 | Coombabah |
| 309031238 | Labrador |
| 309031239 | Paradise Point - Hollywell |
| 309031240 | Runaway Bay |
| 309041241 | Guanaba - Springbrook |
| 309041242 | Tamborine - Canungra |
| 309051243 | Currumbin Valley - Tallebudgera |
| 309051244 | Mudgeeraba - Bonogin |
| 309051245 | Reedy Creek - Andrews |
| 309061246 | Carrara |
| 309061247 | Highland Park |
| 309061248 | Nerang - Mount Nathan |
| 309061249 | Pacific Pines - Gaven |
| 309061250 | Worongary - Tallai |
| 309071251 | Coomera |
| 309071252 | Helensvale |
| 309071253 | Hope Island |
| 309071254 | Jacobs Well - Alberton |
| 309071255 | Ormeau - Yatala |
| 309071256 | Oxenford - Maudsland |
| 309071257 | Pimpama |
| 309071258 | Upper Coomera - Willow Vale |
| 309081259 | Clear Island Waters |
| 309081260 | Merrimac |
| 309081261 | Robina |
| 309081262 | Varsity Lakes |
| 309091263 | Ashmore |
| 309091264 | Molendinar |
| 309091265 | Parkwood |
| 309091540 | Southport - North |
| 309091541 | Southport - South |
| 309101267 | Benowa |
| 309101268 | Bundall |
| 309101269 | Main Beach |
| 309101270 | Surfers Paradise |
| 310011271 | Darra - Sumner |
| 310011272 | Durack |
| 310011273 | Forest Lake - Doolandella |
| 310011274 | Inala - Richlands |
| 310011275 | Oxley (Qld) |
| 310011276 | Wacol |
| 310021277 | Boonah |
| 310021278 | Esk |
| 310021279 | Lake Manchester - England Creek |
| 310021280 | Lockyer Valley - East |
| 310021281 | Lowood |
| 310021282 | Rosewood |
| 310031283 | Brassall |
| 310031284 | Bundamba |
| 310031285 | Churchill - Yamanto |
| 310031286 | Ipswich - Central |
| 310031287 | Ipswich - East |
| 310031288 | Ipswich - North |
| 310031289 | Karalee - Barellan Point |
| 310031290 | Karana Downs |
| 310031291 | Leichhardt - One Mile |
| 310031292 | North Ipswich - Tivoli |
| 310031293 | Raceview |
| 310031294 | Ripley |
| 310031295 | Riverview |
| 310041296 | Bellbird Park - Brookwater |
| 310041297 | Camira - Gailes |
| 310041298 | Carole Park |
| 310041299 | Collingwood Park - Redbank |
| 310041300 | Goodna |
| 310041301 | New Chum |
| 310041302 | Redbank Plains |
| 310041303 | Springfield |
| 310041304 | Springfield Lakes |
| 311011305 | Beaudesert |
| 311021306 | Beenleigh |
| 311021307 | Eagleby |
| 311021308 | Edens Landing - Holmview |
| 311021309 | Mount Warren Park |
| 311021310 | Wolffdene - Bahrs Scrub |
| 311031311 | Boronia Heights - Park Ridge |
| 311031312 | Browns Plains |
| 311031313 | Chambers Flat - Logan Reserve |
| 311031314 | Crestmead |
| 311031315 | Greenbank Military Camp |
| 311031316 | Hillcrest |
| 311031317 | Marsden |
| 311031318 | Munruben - Park Ridge South |
| 311031319 | Regents Park - Heritage Park |
| 311041320 | Greenbank |
| 311041321 | Jimboomba |
| 311041322 | Logan Village |
| 311051323 | Bethania - Waterford |
| 311051324 | Cornubia - Carbrook |
| 311051325 | Loganholme - Tanah Merah |
| 311051326 | Loganlea |
| 311051327 | Shailer Park |
| 311051328 | Waterford West |
| 311061329 | Daisy Hill |
| 311061330 | Kingston (Qld.) |
| 311061331 | Logan Central |
| 311061332 | Rochedale South - Priestdale |
| 311061333 | Slacks Creek |
| 311061334 | Springwood |
| 311061335 | Underwood |
| 311061336 | Woodridge |
| 313011362 | Beachmere - Sandstone Point |
| 313011363 | Bribie Island |
| 313021364 | Burpengary - East |
| 313021365 | Caboolture |
| 313021366 | Caboolture - South |
| 313021367 | Elimbah |
| 313021368 | Morayfield - East |
| 313021369 | Wamuran |
| 313031370 | Kilcoy |
| 313031371 | Woodford - D'Aguilar |
| 313041372 | Burpengary |
| 313041373 | Deception Bay |
| 313041374 | Morayfield |
| 313041375 | Narangba |
| 313041376 | Upper Caboolture |
| 313051377 | Clontarf |
| 313051378 | Margate - Woody Point |
| 313051379 | Redcliffe |
| 313051380 | Rothwell - Kippa-Ring |
| 313051542 | Scarborough - Newport - Moreton Island |
| 314011382 | Albany Creek |
| 314011383 | Cashmere |
| 314011384 | Dayboro |
| 314011385 | Eatons Hill |
| 314011386 | The Hills District |
| 314011387 | Samford Valley |
| 314021388 | Dakabin - Kallangur |
| 314021389 | Murrumba Downs - Griffin |
| 314021390 | North Lakes - Mango Hill |
| 314031391 | Bray Park |
| 314031392 | Lawnton |
| 314031393 | Petrie |
| 314031394 | Strathpine - Brendale |
| 316011413 | Buderim - North |
| 316011414 | Buderim - South |
| 316011415 | Mountain Creek |
| 316011416 | Sippy Downs |
| 316021417 | Aroona - Currimundi |
| 316021418 | Buddina - Minyama |
| 316021419 | Caloundra - Kings Beach |
| 316021420 | Caloundra - West |
| 316021421 | Golden Beach - Pelican Waters |
| 316021422 | Moffat Beach - Battery Hill |
| 316021423 | Parrearra - Warana |
| 316021424 | Wurtulla - Birtinya |
| 316031425 | Coolum Beach |
| 316031426 | Marcoola - Mudjimba |
| 316031427 | Maroochydore - Kuluin |
| 316031428 | Mooloolaba - Alexandra Headland |
| 316051434 | Noosa Heads |
| 316051435 | Noosaville |
| 316051437 | Sunshine Beach |
| 316051438 | Tewantin |
| 316051543 | Peregian Beach - Marcus Beach |
| 316051544 | Peregian Springs |
| 316061439 | Beerwah |
| 316061440 | Caloundra Hinterland |
| 316061441 | Glass House Mountains |
| 316061442 | Landsborough |
| 316061443 | Maroochy Hinterland |
| 316061444 | Palmwoods |
| 316071545 | Bli Bli |
| 316071546 | Diddillibah - Rosemount |
| 316071547 | Eumundi - Yandina |
| 316071548 | Nambour |
| 316081549 | Noosa Hinterland |
| 319031512 | Gympie - North |
| 319031513 | Gympie - South |
| 319031514 | Gympie Region |
