# Supplementary material for: Healthcare services for people with acquired disability in South-East Queensland, Australia: Assessing potential proximity and its association with service obstacles
Source: SSM Popul Health. 2022 Aug 17;19:101209. doi: 10.1016/j.ssmph.2022.101209 (PMC9424535; doi:10.1016/j.ssmph.2022.101209)
Supplement: Multimedia component 2 [file mmc2.docx]

**Supplement 4.** Map of potential access in South-East Queensland to hospitals, including both public and private hospitals. Br = Brisbane, Lo = Logan, Ip = Ipswich, No = Noosa, GC = Gold Coast, Ma = Maroochydore.
