# Supplementary material for: Healthcare services for people with acquired disability in South-East Queensland, Australia: Assessing potential proximity and its association with service obstacles
Source: SSM Popul Health. 2022 Aug 17;19:101209. doi: 10.1016/j.ssmph.2022.101209 (PMC9424535; doi:10.1016/j.ssmph.2022.101209)
Supplement: Multimedia component 4 [file mmc4.docx]

**Supplement 2.** Rehabilitation medicine services mapped.

| **Training setting** |
| --- |
| Brighton Health Campus |
| Cairns Hospital |
| Canossa Private Hospital |
| Eden Private Hospital |
| Gold Coast Private Hospital |
| Gold Coast University Hospital |
| Greenslopes Private Hospital |
| Ipswich Hospital |
| John Flynn Private Hospital |
| Logan Hospital |
| Mackay Base Hospital |
| Mackay Private Hospital |
| Mater Private Hospital Brisbane |
| Princess Alexandra Hospital |
| Queensland Rehabilitation Specialists |
| Redcliffe Hospital |
| Robina Hospital |
| Rockhampton Hospital |
| St Stephen’s Hospital |
| St Vincent’s Private Hospital, Brisbane |
| Sunshine Coast University Hospital |
| Surgical, Treatment and Rehabilitation Services (STARS) |
| The Prince Charles Hospital |
| Townsville University Hospital |
